# Enhancing the Oxidative Stability of Beeswax–Canola Oleogels: Effects of Ascorbic Acid and Alpha-Tocopherol on Their Physical and Chemical Properties

**DOI:** 10.3390/gels11010043

**Published:** 2025-01-07

**Authors:** Sonia Millao, Marcela Quilaqueo, Ingrid Contardo, Mónica Rubilar

**Affiliations:** 1 Department of Chemical Engineering, Faculty of Engineering and Science, Universidad de La Frontera, Temuco 4811230, Chile; marcela.quilaqueo@ufrontera.cl; 2 Scientific and Technological Bioresource Nucleus BIOREN, Universidad de La Frontera, Avenida Francisco Salazar 01145, Temuco 4811230, Chile; 3 Biopolymer Research & Engineering Laboratory (BiopREL), School of Nutrition and Dietetics, Faculty of Medicine, Universidad de los Andes, Chile, Monseñor Álvaro del Portillo 12.455, Las Condes, Santiago 7620086, Chile; icontardo@uandes.cl; 4 Centro de Investigación e Innovación Biomédica (CIIB), Universidad de los Andes, Monseñor Álvaro del Portillo 12.455, Las Condes, Santiago 7620086, Chile

**Keywords:** oleogel, ascorbic acid, alpha tocopherol, oxidative stability, structural properties

## Abstract

The choice of antioxidant to be used in the formulation of an oleogel is crucial to determine its oxidative stability and functionality, as these factors can also affect the physical, chemical, and rheological properties of the oleogel. In this study, the effect of two antioxidants (ascorbic acid, AA, and alpha-tocopherol, AT) and their concentration (0.01, 0.02, and 0.03%) on the physical and chemical properties of beeswax and canola oil oleogels were evaluated. The results show that the type and concentration of antioxidants did not affect the thermal properties of the samples, and in FTIR analyses, no noticeable changes in spectra patterns are observed. Rheological analyses showed that the oleogels containing AA exhibited higher elasticity and resistance to deformation. Accelerated oxidative stability tests (storage at 50 °C and the Rancimat test) showed that AA effectively delayed oxidation. The induction time increased by 2.61-fold at higher concentrations, while AT did not significantly affect oxidation resistance. Overall, it was observed that AA improved oleogel firmness and OBC (up to 1.75-fold and 2.8%, respectively), whereas AT resulted in a softer and less stable gel structure. These results show the importance of antioxidant selection, indicating that hydrophilic antioxidants have promising applications in the formulation of beeswax oleogels.

## 1. Introduction

Oleogels are innovative formulations that consist of structured liquid oils , typically created using oleogelators such as waxes, monoglycerides, and phospholipids. Oleogels have the advantages of solid fats, such as improved eccentric flavor and texture as well as plasticity, without compromising the nutritional composition, and the dietary benefits of liquid vegetable oil [1,2]. Therefore, they have potential applications in the food industry as a healthier substitute for traditional fats such as hydrogenated fats, reducing the need for saturated and trans fats [3,4].

Several types of vegetable waxes have been studied for their ability to form oleogels, including waxes of sunflower, candelilla, carnauba, and rice, as well as beeswax (BW), among others [5]. In particular, oleogels made with BW as a structurant have demonstrated good textural and mechanical properties, so they could be used for various applications in the food industry [6,7]. For example, studies have shown that a 10% concentration of beeswax combined with vegetable oils results in oleogels with desirable rheological properties and a low saturated fatty acid content [8,9] that can be used as substitutes in baked goods, confectionery, dairy, and meat products [9,10]. Research has demonstrated that beeswax-based oleogels are particularly effective at holding large quantities of oil while minimizing the risk of oil exudation, a common issue that can compromise the stability and texture of oleogels. This feature is essential for maintaining the desired consistency and texture over extended periods, making beeswax an ideal candidate for applications where long-term stability and uniformity are important. Furthermore, beeswax’s natural availability, ease of use, and biodegradable nature make it a highly versatile and sustainable choice in oleogel formulation [6,11].

Canola oil is associated with several health benefits [12]. Its regular consumption has been linked to lower LDL cholesterol levels, thus reducing the risk of heart disease. Studies indicate an average reduction in LDL cholesterol of about 16.2% when canola oil replaces saturated fats in the diet [12] because it contains 6–14% α-linolenic acid, 50–60% oleic acid, 50–60% oleic acid, and <7% saturated fatty acids [12,13]. However, as lipid products made from oils, high in unsaturated fatty acids, oleogels are prone to oxidation [14], and the oxidative stability of these oleogels is crucial, as it affects the shelf life and quality of food products incorporating them. In the case of beeswax oleogels, oxidative stability has been studied [15,16] and has been shown to depend on several factors, such as wax concentration, the type of oils used, and the presence of antioxidant compounds such as β-carotene and curcumin [7,17]. Although these compounds have been shown to improve oxidative stability, they may also affect the structural properties of the oleogel depending on the type of structurant used. The antioxidant used could influence the oleogel gelation mechanisms [14,18,19,20].

Alpha-tocopherol (AT) is a lipophilic antioxidant that, in addition to its known ability to inhibit oxidation, which is crucial to extending the shelf life of oleogels [21], is a critical micronutrient for maintaining health and well-being [22]. Conversely, ascorbic acid (AA), a hydrophilic antioxidant, is a potent antioxidant that helps to protect against oxidative stress but can lead to rancidity of oils [23,24]. Ascorbic acid typically demonstrates antioxidant properties in bulk oils. This effect is known as the “polar antioxidant paradox”, which suggests that hydrophilic antioxidants are more effective at reducing lipid oxidation in non-polar environments, such as bulk oil systems [25].

The presence of these antioxidants in oleogels can improve the oxidative stability of the oil, thus prolonging its shelf life, as antioxidants help inhibit or delay the oxidation process in the oil phase and scavenge free radicals, which are responsible for lipid degradation, thus extending the shelf life of the product. However, reports on oleogel oxidation are still in the exploratory stage, so antioxidants have become an important issue to address [26,27], as their incorporation in oleogels requires an understanding of the compatibility between structuring agents, the type of antioxidants and oils used, and their concentration to ensure stability and efficacy according to the specific application and desired shelf life of the product.

This research evaluated the effect of the antioxidants AT and AA in oleogels prepared with canola oil and BW on their physical and chemical properties. Changing the type and concentration of antioxidants in the preparation of oleogels, besides affecting oxidative stability, could also affect the mechanical and rheological stability of oleogels.

## 2. Results and Discussion

### 2.1. X-Ray Diffraction

**Figure 1:**
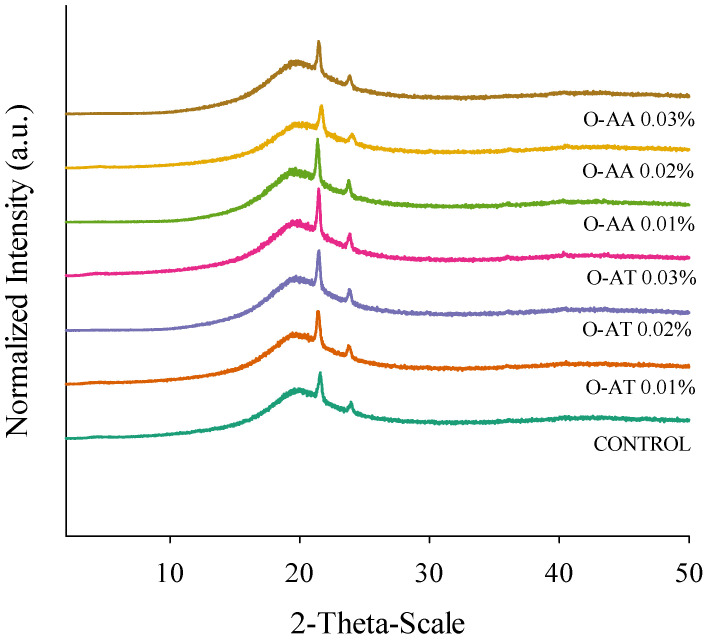
X-ray diffraction patterns of oleogels prepared with different antioxidants (AA: ascorbic acid; AT: alpha-tocopherol) and concentrations (0.01, 0.02, and 0.03%).

X-ray diffraction can be used to analyze the internal structure of materials. The diffraction pattern obtained showed that adding AA and AT did not change the crystallinity of oleogels, since the patterns showed that the diffraction peaks of all samples were almost the same (Figure 1). The pattern of the control oleogel showed that the sample is a semi-crystalline solid with a medium degree of crystallinity, while the amorphous component contributed a large proportion. The strong diffraction peaks at 19, 21, and 23°, corresponding to d spacing of 4.5, 4.1, and 3.7 Å, respectively. These are distinctive peaks of BW, reflecting the presence of β, α, and β’ crystals within the structure [28].

### 2.2. Infrared Spectroscopy (FTIR)

**Figure 2:**
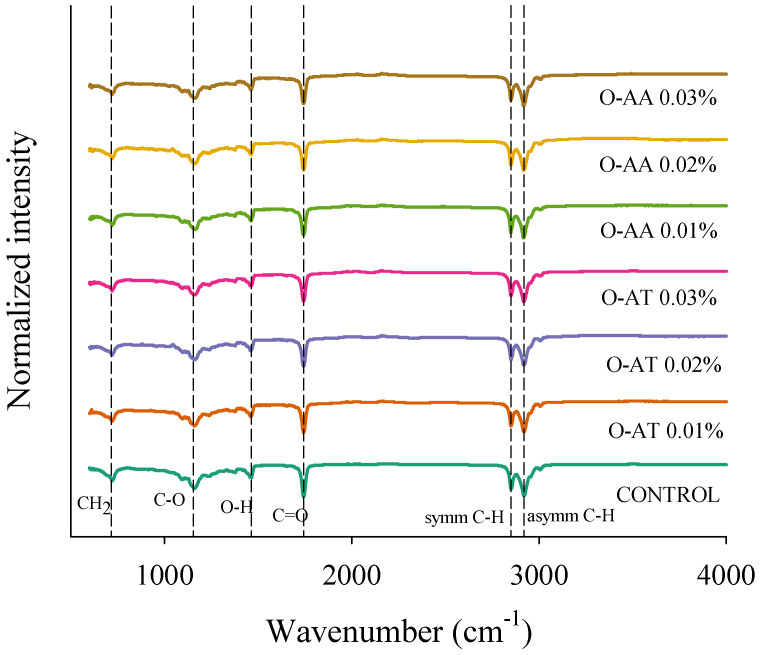
FTIR spectra of oleogels prepared at different antioxidants (O-AA: oleogel with ascorbic acid; O-AT: oleogel with alpha-tocopherol) and concentrations (0.01, 0.02, and 0.03%).

The FTIR spectra of the oleogels (Figure 2) provide information on possible molecular interactions between the components of the mixture. As the overlaid spectra show, all samples exhibited similar absorption bands. No evident shifts or new peaks from interactions between the components of the phases were detected when the antioxidants AA and AT were incorporated, indicating that they do not give rise to new chemical bonds or significant structural alterations at the molecular level.

Three distinct primary spectral bands can be seen in the spectra. Peaks around 2850 and 2920 cm^−1^ were attributed to symmetric and asymmetric CH stretching of the alkane and hydrocarbon groups of canola oil and BW [29]. In addition, a prominent peak was identified around 1742 cm^−1^ that can be attributed to the C=O stretching vibrations of esters and free fatty acids resulting from the overlapping combination between the oil and beeswax [11,29]. The band with a peak around 1157 cm^−1^ can be attributed to the stretching of the C–O bonds of the aliphatic esters and CH_2_ bending vibrations, and the peak around 1459 cm^−1^ corresponds to the O–H bending of the carbonyl group. The band with a peak around 719 cm^−1^ can be attributed to the CH_2_ rocking of the beeswax hydrocarbons [29].

In the FTIR spectra of the oleogels with AA, significant changes (*p* < 0.05) were observed in the position of the absorption band associated with the CH_2_ groups between the control oleogel and the samples with AA, shifting from 1239 to 1241 cm^−1^ and 1460 to 1463 cm^−1^ as a function of increasing ascorbic acid concentration, demonstrating the contribution of the CH_2_ groups from this antioxidant. Furthermore, a decrease in the position of the band (wavenumber) attributed to the C=O stretching vibrations of the carbonyl group in the esters of triglycerides was observed from 1744 to 1743 cm^−1^, suggesting that hydrogen bond interactions could be promoted between the carbonyl groups of canola oil and AA in the oleogel. However, the concentrations of this antioxidant used were very low; therefore, there was a dilution effect on the oleogels, and the greater interactions of the system could be attributed to van der Waals forces. Although AA exhibited a peak indicative of the OH stretching modes (Figure S1), no peaks were observed in the oleogels in the spectral range of 3900–3050 cm^−1^. Similar results were observed for Dhal et al. [30] and Chaturvedi et al. [31]. In the case of oleogels treated with AT, the main shifts in the absorption band position were also observed from 1460 to 1463 cm^−1^, associated with the contribution of C=C stretching in the phenyl skeletal and methyl asymmetric bending of AT [32], and a decrease from 1744 to 1743 cm^−1^ was observed. Similarly to AA, the AT oleogels did not exhibit significant peaks assigned to O–H stretching (Figure S1).

### 2.3. Differential Scanning Calorimetry (DSC)

**Figure 3:**
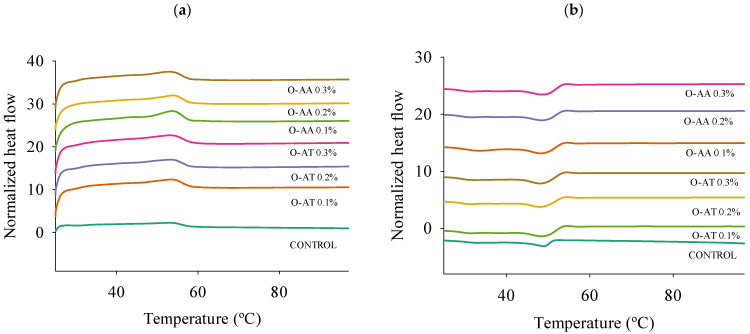
Normalized DSC thermograms of oleogels with ascorbic acid (AA) and alpha-tocopherol (AT) as antioxidants at different concentrations (0.01%, 0.02%, and 0.03%) are shown for the heating (**a**) and cooling (**b**) phases.

The thermal behavior of oleogels based on different types and concentrations of antioxidants was characterized by DSC (Figure 3). According to Table 1, two peak melting (T_pm_) (at 35.36–38.08 and 53.86–53.92 °C) and peak crystallization (T_pc_) (at 49.25–49.44 and 30.92–32.75 °C) temperatures were observed. The oleogels presented a dual structure formed by the different BW fractions [29,33]. Although there were differences of approximately 2 °C, particularly in T_pm1_ and T_pc2_, for the different oleogels studied, in general, no significant differences (*p* < 0.05) were found in the T_m_ and T_c_ of the oleogels according to the type and concentrations of the antioxidants evaluated. This suggests that the antioxidants did not significantly intervene in the interaction of BW with the oily phase in both the melting and crystallization processes.

### 2.4. Optical Microscopy

**Figure 4:**
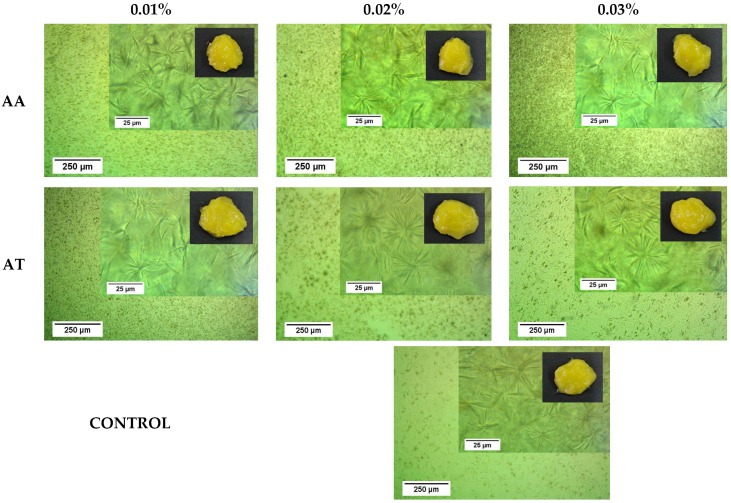
Optical microscopy images and visual appearance of oleogels prepared with ascorbic acid (O-AA) and alpha-tocopherol (O-AT) as antioxidants at different concentrations (0.01, 0.02, and 0.03%).

Figure 4 shows the crystal microstructure of O-AA and O-AT at different concentrations and how these antioxidants affect the spatial distribution and, thus, the homogeneity of the oleogel microstructures. When comparing O-AA and O-AT with the control, it can be observed that AA caused the distribution of the needle crystals in the internal structure of the system to be more homogeneous and dispersed in the matrix; this could cause a more significant amount of liquid oil to be trapped in the crystal network, and the gel structure to become more stable and firmer [14]. In contrast, in O-AT, the needle crystals form more groups and aggregate, leading to less homogeneity in the distribution of these groups.

### 2.5. Oil Binding Capacity (OBC)

**Figure 5:**
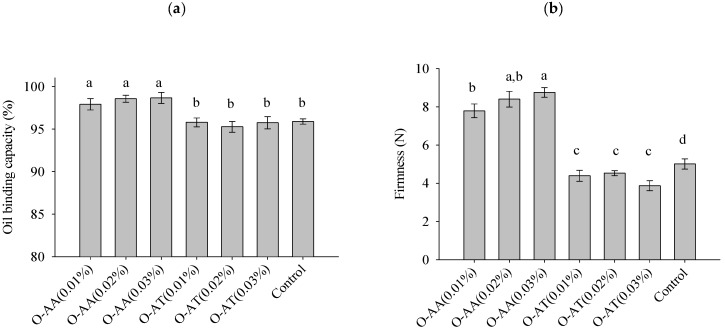
Oil binding capacity (**a**) and firmness (**b**) of oleogels prepared with different antioxidants (O-AA: oleogel with ascorbic acid; O-AT: oleogel with alpha-tocopherol) and concentrations (0.01, 0.02, and 0.03). The lowercase letters a–d represent significant differences determined by Tukey’s test (*p* < 0.05).

The OBC allows for assessing the capacity of the beeswax crystalline structure to trap the oil phase [7]. The OBC values of oleogels range between 94.74 ± 0.72% and 95.26 ± 0.63% for O-AT and between 97.90 ± 0.66 and 98.65 ± 0.64% for O-AA (Figure 5a). According to these values, there was a significant difference between O-AA and O-AT, i.e., between antioxidant types, but their different concentrations did not significantly affect the OBC. The significantly higher OBC for O-AA suggests that the crystalline structure formed by the BW created a more homogeneous physical barrier, as can be corroborated in the optical microscopy images (Figure 4). This behavior increases stability and reduces oil loss, restricting the flow of liquid oil and thus forming a more stable oleogel [34,35].

### 2.6. Firmness

As shown in Figure 5, the incorporation of AA in oleogels significantly increased the firmness (Figure 5b) and OBC (Figure 5a) concerning oleogels with AT, demonstrating higher stability, which is essential to maintain the desired texture. In addition, increasing the ascorbic acid concentration also increased firmness, although this increase is only significant (*p* < 0.05) when increasing the concentration from 0.01 to 0.03% (Figure 5b). The presence of hydroxyl groups in the hydrophilic antioxidants probably allowed the formation of hydrogen bonds with the gel matrix. This bonding may improve the structural integrity of the oleogel, contributing to a denser and more stable network [3,18].

On the other hand, AT had the opposite effect. As the AT concentration increased, oleogel firmness decreased, although these variations were only significant when alpha-tocopherol concentration increased from 0.01 to 0.03%. Hydrophilic antioxidants may interact differently from lipophilic antioxidants, affecting the overall physical and chemical properties of oleogels [6]. The decrease in the hardness of the oleogels suggests that the AT interfered with the gelling action of the BW interacting with lipid molecules and influenced the formation of the crystalline network beeswax creates when mixed with oils.

Beeswax esters are crucial for structuring oleogels, and AT may interact with them, potentially disrupting their capacity to create binding sites. Behavior like that obtained by Barragán Martínez et al. [29] when incorporating β-carotene into BW/canola oil oleogels, where it possibly interacted with the BW components and/or the fatty acids of canola oil, promotes the softening of the oleogels.

### 2.7. Rheological Properties

**Figure 6:**
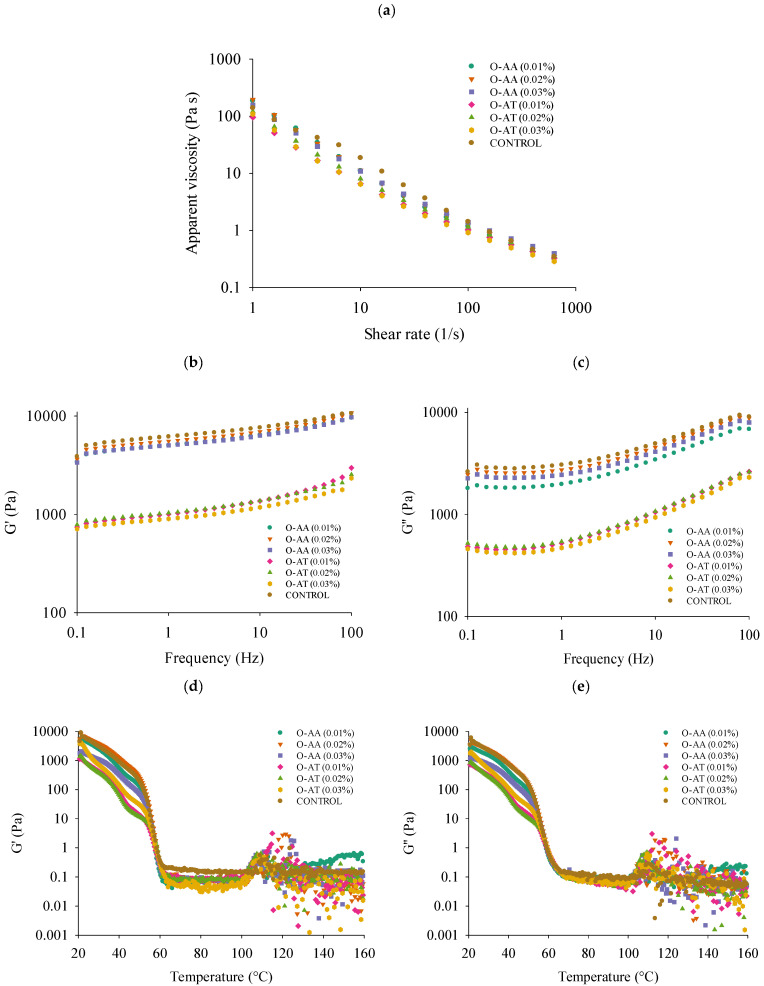
Rheological properties of oleogels with different antioxidants (O-AA: oleogel with ascorbic acid; O-AT: oleogel with alpha-tocopherol) and concentrations (0.01, 0.02, and 0.03%). Apparent viscosity (Pa·s) over shear rate (**a**). Changes in the viscoelastic properties G′ (**b**) and G″ (**c**). Thermodynamic properties (20 a 160 °C) (**d**,**e**).

The rheological analysis of the oleogels with antioxidants at different concentrations was performed to evaluate the sweep of viscosity, elastic modulus G’, and viscous modulus G’’ as a function of shear rate and frequency, respectively (Figure 6). Viscosity decreases as the shear rate increases (Figure 6a), suggesting non-Newtonian fluid behavior, particularly shear thinning behavior [27,36]. The oleogels prepared with AA and AT at different concentrations showed similar viscosities and stable falling behavior in a shear rate range between 1 and 1000 s^−1^. However, an effect on the magnitude of the apparent viscosity was observed, which is in agreement with the OBC and firmness results (Figure 5a,b), where weaker gels were obtained with the addition of AT.

The same behavior was observed in frequency sweep tests, where oleogels with AA show higher storage modulus (G′) (Figure 6b) than oleogels with AT. This suggests improved resistance to deformation when external forces are applied, implying that the gels are stronger. AA is a molecule that provides ionic charge to the material, which could modify the electrostatic balance and affect the aggregation–disaggregation capacity of the lipids, altering the viscoelastic behavior of the oleogels. Talló et al. [37] indicated that charge of lipid particles is essential for the effective formation of oleogels and that the structural and viscoelastic properties of oleogels change based on the charge ratio of the lipid molecules. However, all the oleogels showed similar behavior to solids in the frequency range from about 1 to 10 Hz, characterized by higher values of the G′ than the G″ (Figure 6b,c). This indicates that oleogels have an elastic component essential to maintaining their structure during processing and application. This behavior is independent of the type and concentration of the antioxidant used. Furthermore, at higher frequencies near 100 Hz, the moduli converge, indicating that the gel′s viscous behavior (G″) becomes dominant. This marks the flow point, where G′ and G″ are equal, causing the sample to transition to a flowing state [38].

Temperature sweep tests were performed to evaluate the overall gelation behavior. For all oleogels, the heating phase induced a continuous reduction in the storage and loss moduli (Figure 6d,e) up to about 60 °C. This means that the BW oleogel’s structure remained relatively uniform up to this temperature. However, AT had a greater impact on the viscoelastic properties than AA, confirming that oleogels with AT were weaker. This behavior was maintained when the temperature was increased to <60 °C. From 60 °C onwards, both the G′ and G″ of all oleogels slowed down, indicating a weakening of the oleogels, i.e., the system started to undergo a phase transformation, with the oleogels fully converting into a liquid state during the continued heating process [18,27]. Above 100 °C, a fluctuation in the G′ and G″ responses was observed, confirming the molten state of the crystals and liquid-like behavior of the oleogel, which collapsed. In addition, these fluctuations reflect the thermal sensitivity of the liquid state of the oleogels at high temperatures.

### 2.8. Oxidative Stability

**Figure 7:**
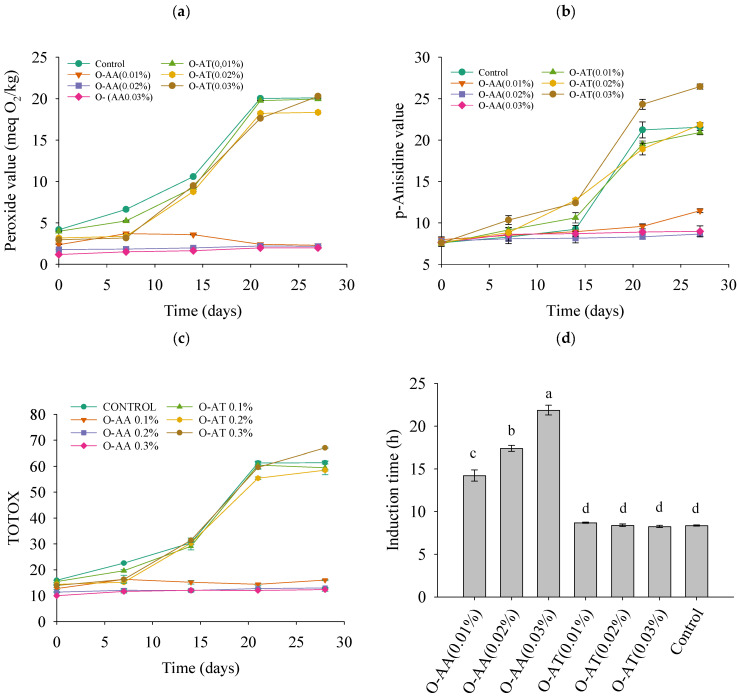
Accelerated oxidative stability: peroxide value (**a**); *p*-anisidine value (**b**); TOTOX (**c**) after 28 days of storage at 50 °C; and induction time (IT) (**d**) of oleogels prepared with different antioxidants (O-AA: oleogel with ascorbic acid; O-AT: oleogel with alpha-tocopherol) and concentrations (0.01, 0.02, and 0.03%). The lowercase letters a–d represent significant differences determined by Tukey’s test (*p* < 0.05).

The oxidative stability of O-AA and O-AT (Figure 7) was evaluated by measuring primary oxidation (POV), secondary oxidation (*p*-AV), and total oxidation (TOTOX). In addition, the induction time (IT) was determined by the Rancimat test.

After 28 days of storage at 50 °C, the PV of O-AA did not exceed 4 (meq O_2_/kg) and had a slow growth rate. In contrast, on day 14, the PV of O-AT for all three concentrations was close to 10 (meq O_2_/kg), sharply increasing the oxidation growth rate (propagation phase) (Figure 7a). For pAV, the trend of the oxidation curve was similar to that of PV (Figure 7b). This suggests that AA effectively protected the oleogel against lipid oxidation by slowing down the propagation phase of oxidation and reducing the production of secondary oxidation compounds. These compounds, measured by *p*-AV, include aldehydes and ketones formed through the further oxidation of hydrogen peroxide, with some degree of hysteresis observed [18]. In addition, TOTOX (Figure 7c) was calculated for all oleogels based on the PV and *p*-AV results. The addition of AA reduces the rate of increase in TOTOX, and the lower the TOTOX, the higher the quality of oleogels, which could extend their shelf life.

This can be corroborated by comparing IT, where AA significantly (*p* < 0.05) increased IT values compared to the control (8.37 ± 0.07 h) by 1.7-fold at the 0.01% concentration, 2.1-fold at the 0.02% concentration, and 2.61-fold at the 0.03% concentration.

This could be due to the hydrophilic antioxidant’s inability to dissolve in the oil phase fully. Instead, it dispersed on the surface and interstitium of the oleogels, where it is more susceptible to oxygen radicals in the air and is oxidized first, since one of the antioxidant mechanisms of ascorbic acid is the scavenging of molecular oxygen [39], thus playing a decisive role in delaying the oxidation of oleogels [18]. AT had no significant effect (*p* > 0.05) on IT with respect to the control at any of the concentrations studied (8.70 ± 0.07 h at 0.01%; 8.40 ± 0.15 h at 0.02% and 8.26 ± 0.66 h at 0.03%). This may be due to the reduced stability of the gel network and the release of oil caused by extended storage at elevated temperatures (50 °C) [27].

## 3. Conclusions

The antioxidants were dispersed in the oil phase of oleogels without changing the melting characteristics and crystallinity of the oleogels. In FTIR analyses, no noticeable changes in spectra patterns were observed after antioxidant addition. However, due to the low antioxidant concentrations, it is likely that Van der Waals forces will dominate the possible interactions.

Therefore, this work showed that AA and AT can modify the firmness and OBC of canola oil and BW oleogels. AA increased oleogel firmness and OBC up to 1.75-fold and 2.8%, respectively, particularly at higher concentrations (0.03%), indicating that even lower concentrations of the structurant could effectively stabilize the oils when combined with this antioxidant. In contrast, AT showed a softening effect on the gel structure.

Rheological tests indicated that oleogels with AA exhibited higher elasticity (storage modulus G′) and viscosity, reflecting stronger gels, and stability tests showed that AA effectively delayed lipid oxidation and prolonged the induction time, indicating its superior antioxidant capacity compared to AT.

This study provides valuable information on the role of antioxidants in oleogel systems, highlighting AA as a more effective stabilizer for both gel structure and oxidative stability. Future research could focus on optimizing hydrophilic antioxidant concentrations and the combined effects with lipophilic antioxidants.

## 4. Materials and Methods

### 4.1. Materials

Cold-pressed canola oil was used, sourced from Alimentos Soy Saludable SPA (Osorno, Chile). Beeswax was purchased from Sigma-Aldrich (Santiago, Chile). Alpha-tocopherol, ascorbic acid, and analytical grade chemicals, such as KOH, KI, Na_2_S_2_O_3_, and *p*-anisidine, were acquired from Sigma-Aldrich Ltd. (Steinheim, Germany).

### 4.2. Preparation of Oleogels with Antioxidants

BW oleogels were prepared using methods from previous research, with modifications [40]. Batches of 100 g were prepared with canola oil using a BW concentration of 10%. Oleogel prepared with AT (O-AT). The AT was incorporated directly into the oil phase, stirring the mixture for 1 h in the dark. Oleogels prepared with AA (O-AA). First, the ascorbic acid was dissolved in methanol (99.9%). Methanol was used as a solvent to dissolve the hydrophilic antioxidant and to allow its incorporation into the oil phase, ensuring uniform distribution. The solution was stirred for one hour in the dark, and then the methanol was evaporated with nitrogen gas.

Antioxidants were incorporated into the oleogels at 0.01, 0.02, and 0.03% concentrations. Finally, the mixture (oil phase and structurant) was heated on a hot plate at 70 °C, with magnetic stirring at 300 rpm for 30 min, and covered with aluminum foil. The oleogels were allowed to cool to room temperature for at least 2 h and refrigerated at 6 °C. An oleogel without antioxidants was used as a control. The samples were analyzed after 24 h under refrigeration.

### 4.3. X-Ray Diffraction (XRD)

The XRD analysis was performed using a D8 Advance diffractometer (Bruker, Karlsruhe, Germany) equipped with a CuKα1,2 and a Kβ X-ray tube, operating at 40 kV and 40 mA. The oleogels were positioned on a Lucite sample holder and gently compressed by hand. Data scans were recorded at 2θ = 2–50° with a step size of 0.02° and a dwell time of 1 s per step. The data obtained were processed and analyzed using EVA software (Bruker Corporation, Billerica, MA, USA).

### 4.4. Fourier Transform Infrared (FTIR) Spectroscopy

The FTIR analyses of the samples were performed using an ATR-FTIR Spectrum One spectrometer (Agilent, Cary 6390, NC, USA). The samples were placed on the ATR diamond crystal and clamped using pressure and were examined at room temperature within a spectral range of 4000 to 600 cm^−1^, using a resolution of 4 cm^−1^ and conducting 30 scans per sample. All measurements were carried out in triplicate. The spectra were processed using OriginPro 2019b (OriginLab Corporation, Northampton, MA, USA). This included baseline adjustment and normalization of the relative transmittance.

### 4.5. Optical Microscopy Analyses

Optical microscopy was used to analyze the microstructure of the oleogels. An optical microscope from Euromex (Euromex Microscope B.V., Diuve, The Netherlands) was utilized, equipped with 10× and 100× objective lenses for image capture.

### 4.6. Differential Scanning Calorimetry (DSC) Analysis

The DSC analysis of the oleogels was assessed using a DSC 1 STAR (Mettler-Toledo, Greifensee, Switzerland). A sample weighing 20–25 mg was placed in the pan, with an empty pan as the reference. Nitrogen gas was used for purging. The samples were heated from 25 to 150 °C and subsequently cooled to 25 °C at a rate of 5 °C/min. The STARe (DB V12.10, Mettler-Toledo, Greifensee, Switzerland) software determined the peak melting and crystallization temperatures. The analysis was carried out in triplicate.

### 4.7. Oil Binding Capacity (OBC) Analyses

The OBC was employed using the methodology described by Blake and Marangoni [41], with modifications. The test was conducted using a Neofuge 15R centrifuge (Heal Force, Shanghai, China), where samples were centrifuged at 6888 rcf for 40 min at 4 °C. Approximately 1 g of the sample was sieved, and the released oil was subsequently discarded. The percentage of *OBC* was then calculated using Equation (1).

(1) $$OBC\;\left(\%\right)=1-\frac{m_{i}-m_{f}}{m_{i}}\times 100$$

where *m_i_* represents the sample’s weight before centrifugation and *m_f_* denotes the initial weight.

### 4.8. Firmness Analyses

The firmness of the oleogels was measured using a TA.XT PlusC texture analyzer (Stable Micro System, Surrey, UK) equipped with a 5 kg load cell. In this process, 30 g of the sample (3 cm in height and 4 cm in diameter for all samples) was transferred to a 50 mL beaker and kept at 4 °C for 24 h. The samples were taken out of storage immediately before testing. A cylindrical probe with a diameter of 5 mm was employed to penetrate the samples to a depth of 10 mm at a steady speed of 1 mm/s. The peak positive force, measured in newtons (N), was recorded as an indicator of the sample’s firmness [40].

### 4.9. Rheological Analysis

The rheological characteristics of the oleogels were evaluated according to the methodology described by Millao et al. [25]. A rheometer (Discovery HR2, TA Instruments, New Castle, DE, USA) was used. The samples were sealed with a solvent trap throughout the analysis to maintain a consistent temperature throughout the system. The data were processed using the TRIOS software (version 5.1.1, TA Instruments, New Castle, DE, USA). The flow properties were evaluated at 25 °C, with a shear rate ranging from 1 to 1000 (1/s). The apparent viscosity (Pa·s) was recovered.

The viscoelastic properties of the samples were assessed under oscillatory conditions at 1 Hz, with strain values ranging from 0.001 to 20% at 25 °C. The elastic modulus (G′) and the viscous modulus (G″) were evaluated.

Frequency sweep test was conducted with the temperature maintained at 25 °C. The moduli responses (G′ and G″) to increasing frequencies (ranging from 0.1 to 100 Hz) at a strain of 0.01% within the linear viscoelastic range were evaluated. Temperature sweeps test evaluated the thermal stability of the oleogels over a temperature range of 20 to 160 °C, using a linear heating rate of 5 °C/min, a shear strain of 0.01%, and a frequency of 1 Hz.

### 4.10. Oxidative Stability Analysis

To evaluate the oxidative stability of the oleogels, the samples were kept at 50 °C in a dark environment for 28 days of storage, and peroxide (PV), p-anisidine (pAV), and TOTOX values were determined at days 0, 7, 14, 21, and 28 according to Millao et al. [25]. In addition, the Rancimat test, an accelerated method to evaluate the oxidative stability of oleo-gels, was employed [42].

The method outlined in the AOCS Recommended Official Method Cd 8-53 was followed for the PV analysis. In summary, 5 g of the oleogel was dissolved in a 30 mL acetic acid solution with chloroform (3:2). Then, 0.5 mL of a saturated KI solution was added, followed by 30 mL of distilled water. Afterward, 1 mL of 1% starch solution was added. Finally, it was titrated with sodium thiosulfate (0.01N). The PV was calculated using Equation (2).

(2) $$V=\frac{S\times N\times 1000}{w}$$

where *S* refers to the volume of sodium thiosulfate in milliliters (corrected for the blank), *N* represents the normality of the sodium thiosulfate solution, and *w* denotes the sample weight in grams.

The pAV (secondary oxidation) was determined following the official AOCS Cd 18–90 method described by Mohanan et al. [43]. A 0.25% p-anisidine standard solution was prepared using glacial acetic acid. A 5 mL aliquot of the sample in isooctane (1 g/25 mL) plus 1 mL of the p-anisidine standard solution were mixed in another tube. The mixture was left in darkness for 10 min. Then, the absorbance of solutions + sample (*A_1_*), sample + p-anisidine (*A_2_*), and isooctane + p-anisidine (*A_3_*) was measured at 350 nm by means of UV-vis spectrophotometry (Synergy HT, BioTek Instruments Inc., Winooski, VT, USA), in triplicate. Equation (3) was used to calculate *pAV*.

(3) $$pAV=25\times [(1.25\times \left(A_{3}-{(A}_{1}-A_{2}\right)]/w$$

where *w* represents the grams of sample.

The *TOTOX* value of the oleogels was determined to provide an overall assessment of their oxidation level. The *TOTOX* value was calculated using Equation (4).

(4) TOTOX=pAV+2pV

where *pAV* is the p-anisidine value, and *pV* is the peroxide value.

The Rancimat test was employed as an accelerated method for evaluating the oxidative stability of edible oils and fats [42]. The 743 Rancimat (Metrohm, Herisau, Switzerland) was used for the analysis. In this process, 5 g of the oleogel was placed in a closed circuit, exposed to air, and heated to 110 °C with an airflow rate of 20 mL/h. The analysis was performed in triplicate. The induction time (IT) was recorded, marking the onset of volatile compound production.

### 4.11. Statistical Analysis

An analysis of variance (ANOVA) was performed with a significance level of 0.05. In cases where significant differences were found, Tukey’s test was performed. In addition, a multiple comparison procedure was performed on all samples of FTIR. The specific positions of the bands (wavenumbers) of the FTIR spectra for each sample were processed, and the significance of the differences was tested at a 95% confidence level using Fisher’s Least Significant Difference (LSD). All statistical analyses were performed using the Statgraphics Centurion XIX software (Version 19.6.03, Manugistics, Inc., Rockville, MD, USA). 

## Figures and Tables

**Table 1:** Thermal properties of oloegels prepared with different antioxidants and concentrations.

Sample	Heating		Cooling	
	T_pm1_	T_pm2_	T_pc1_	T_pc2_
	(°C)	(°C)	(°C)	(°C)
O-AA (0.01%)	37.29 ± 4.30 ^a^	53.92 ± 0.36 ^a^	49.31 ± 0.12 ^a^	32.75 ± 0.44 ^a^
O-AA (0.02%)	38.08 ± 2.40 ^a^	53.70 ± 0.46 ^a^	49.44 ± 0.13 ^a^	32.11 ± 0.48 ^a^
O-AA (0.03%)	35.64 ± 2.10 ^a^	53.84 ± 0.38 ^a^	49.30 ± 0.38 ^a^	32.39 ± 0.48 ^a^
O-AT (0.01%)	36.17 ± 1.39 ^a^	53.97 ± 0.27 ^a^	49.33 ± 0.22 ^a^	31.69 ± 0.13 ^a^
O-AT (0.02%)	36.14 ± 1.63 ^a^	53.86 ± 0.34 ^a^	49.30 ± 0.21 ^a^	30.92 ± 0.44 ^a^
O-AT (0.03%)	35.36 ± 0.38 ^a^	53.86 ± 0.34 ^a^	49.25 ± 0.30 ^a^	32.14 ± 0.19 ^a^
Control	33.94 ± 1.50 ^a^	53.47 ± 0.27 ^a^	49.06 ± 0.10 ^a^	32.66 ± 0.29 ^a^

O-AA: oleogels with ascorbic acid; O-AT: oleogels with alpha-tocopherol; T_pm (1–2)_, peak melting; T_pc (1,2,)_, peak crystallization. Same letters in the same column indicate no significant difference between antioxidant type and concentrations (*p* < 0.05).

## Data Availability

The raw data supporting the conclusions of this article will be made available by the authors on request.

## Supplementary Materials

The following supporting information can be downloaded at: https://www.mdpi.com/article/10.3390/gels11010043/s1, Figure S1: FTIR spectra of beeswax (BW), canola oil, ascorbic acid (AA) and alpha-tocopherol (AT).
