# Supplementary figures and images for: Enhancing the Oxidative Stability of Beeswax–Canola Oleogels: Effects of Ascorbic Acid and Alpha-Tocopherol on Their Physical and Chemical Properties
Source: Gels. 2025 Jan 7;11(1):43. doi: 10.3390/gels11010043 (PMC11765113; doi:10.3390/gels11010043)

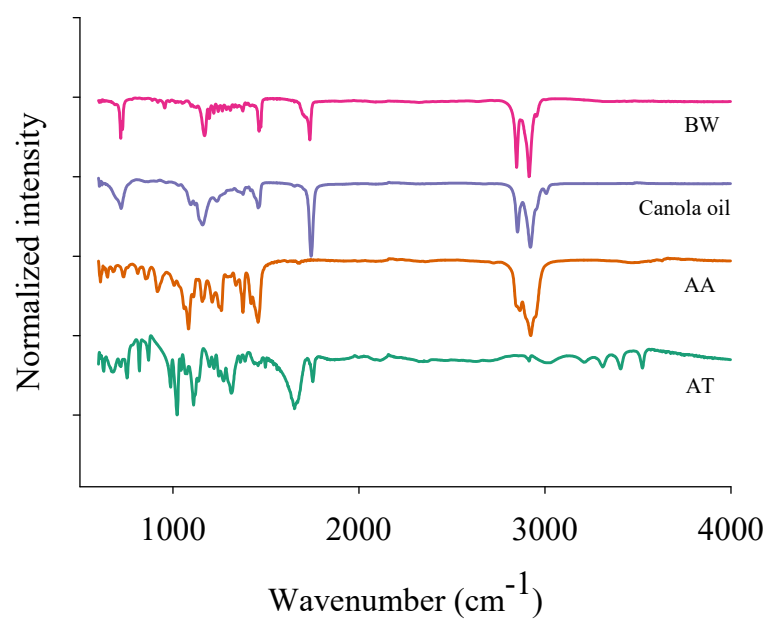

**Figure S1:** FTIR spectra of beeswax (BW), canola oil, ascorbic acid (AA) and alpha-tocopherol (AT).

Supplement: Supplementary file 1 [file gels-11-00043-s001.zip › gels-3365785-supplementary.pdf]
